# M2 Macrophage Derived Extracellular Vesicle-Mediated Transfer of MiR-186-5p Promotes Colon Cancer Progression by Targeting DLC1

**DOI:** 10.7150/ijbs.69405

**Published:** 2022-02-07

**Authors:** Junfeng Guo, Xingyu Wang, Qingdong Guo, Shengtao Zhu, Peng Li, Shutian Zhang, Li Min

**Affiliations:** Department of Gastroenterology, Beijing Friendship Hospital, Capital Medical University, National Clinical Research Center for Digestive Disease, Beijing Digestive Disease Center, Beijing Key Laboratory for Precancerous Lesion of Digestive Disease, Beijing, 100050, P. R. China

**Keywords:** Colon cancer (CC), M2 macrophage, Extracellular vesicles (EVs), miR-186-5p, DLC1

## Abstract

Colon cancer (CC) is one of the most common malignances in digestive tract. M2-polarized macrophages within the tumor microenvironment could facilitate CC cell growth by transferring molecules via extracellular vesicles, but the mechanisms are not fully elucidated. The current study aims to identify the possible effectors in M2 macrophage-derived extracellular vesicles (M2-EVs) and reveal related molecular mechanisms. In our study, we validated the promotion effects of M2-EVs on the proliferation and motility of CC cells, which was found to be dependent on the EVs enclosed molecules by a mild EVs digestion assay. Then we found that miR-186-5p was enriched in M2-EVs and was responsible for the tumor promoting functions of M2-EVs. Furthermore, mechanism investigation revealed M2-EVs transferring miR-186-5p inhibited DLC1 expression by targeting its 3'UTR, and restored DLC1 successfully neutralized the tumor-promoting effects of M2-EVs transferring miR-186-5p via inhibiting the β-catenin pathway. Our study revealed that M2-EVs facilitates the growth and motility of CC cells by delivering the enclosed miR-186-5p, which directly targets DLC1 mRNAs and facilitates their degradation, which could provide a potential biomarker and therapeutic target for CC.

## Introduction

With an estimation of 1,148,515 new cases and 576,858 deaths in 2020 worldwide, colon cancer (CC) becomes one of the most commonly diagnosed malignant cancer types 1, and the incidence of CC is still rising especially in developing countries 2. Recently, the intercellular interactions among cancer cells and other cells in the tumor microenvironment (TME) attract immense attention 3, since each step of oncogenesis and progression is largely affected by the surrounding stromal cells 4.

Tumor-infiltrating macrophage is an abundant component in the TME with high remodeling abilities, which can be polarized into two main subtypes: M1 phenotype with pro-inflammation and anti-tumor effects; M2 phenotype with anti-inflammation and pro-tumor effects 5. It is commonly recognized that most macrophages infiltrated into tumors are M2-polarized, which are also named tumor-associated macrophages (TAMs) 6. Extracellular vesicles (EVs) are enriched in the TME and play a critical role in the communication and exchange of materials between cancer cells and their surrounding stromal cells 7. Numerous studies had demonstrated that TAM-derived EVs mediate cellular communication with tumor cells by transferring macromolecules, therefore enhanced tumor progression in various types of malignancies 8-10. Those studies proposed a series of EVs-associated bioactive molecules (e.g., LncRNA, microRNA, and protein) with detailed mechanisms. However, none of those studies verified the origin of these molecules, and whether the RNAs or the proteins, located on the surface or inside the TAM-EVs, contribute most to the tumor-promoting effects is still controversial.

Additionally, nearly all studies on TAM-derived EVs focused on tumor tissue isolated TAMs 11, 12. However, even though the M2 phenotype is considered to be dominant in TAMs, many studies have proved that TAMs isolated from tumor tissue are a mixture of M1 and M2 13, 14. Thus, the heterogeneity of tumor tissue isolated TAMs would also largely mask the tumor-promoting mechanism of their EVs 15.

In this study, we focused on *in vitro* interleukin-induced M2 macrophage, which would be a homogeneous subgroup, and proved that M2 macrophage-derived EVs promoted cancer growth and motility by delivering the enclosed molecules. Furthermore, we confirmed that miR-186-5p is enriched in M2-EVs and could promote the growth and motility of CC cells via targeting DLC1 through the delivery of EVs. Thus, we not only revealed a new mechanism of EVs transferring miR-186-5p from M2 to CC cells, but also proposed a potential therapeutic target for cancer treatment.

## Materials and methods

### Cell lines and cell culture

Human CC cell lines (SW480 and HCT-8) and monocyte THP-1 cells were obtained from Cell bank of Chinese Academy of sciences. RPMI 1640 medium with 10% Gibco fetal bovine serum (Thermo Fisher, cat. 10100147, Waltham, MA, USA) was used to culture all those three cell lines, at 37°C with 5% CO_2_ and optimal humidity consistently. Cells in our experiments were authenticated for STR analysis, exhibited a negative result for mycoplasma contamination. THP-1 cells were treated with 100ng/ml phorbol 12-myristate 13-acetate (Sigma-Aldrich, cat. 79346, St. Louis, MI, USA) for 24h for differentiation into M0 macrophages 16, then 20ng/ml IL-4 (Solarbio Science & Technology, cat. P00133, Beijing, China) plus 20ng/ml IL-13 (Solarbio Science & Technology, cat. P00131, Beijing, China) were used to stimulate M0 macrophages into M2 macrophages after 48h 17.

### Isolation of EVs and protein quantification

M0 and M2 macrophages were cultured with 10% EVs-free FBS, conditioned medium was gathered after 48h and cell debris was removed by centrifugation (2500g for 30min and 8500g for 30min), the supernatant was collected and centrifuged at 120 000×g for 70min twice, the pellets were then washed by PBS, finally resuspended in PBS and stored at -80°C 18, 19. The centrifugation procedures were proceeded under 4°C using Hitachi ultracentrifuge CS150FNX (Hitachi, Tokyo, Japan). Pierce BCA Protein Assay Kit (Thermo Fisher, cat. 23225, Waltham, MA, USA) was used for the quantification of the proteins, using stand curve at the absorbance of 562nm 19.

### Labeling and characterization of EVs

PKH67 Kit (Sigma-Aldrich, cat. PKH67GL, St. Louis, MI, USA) was used for the labeling of EVs. 100uL EVs were mixed with 0.4uL PKH67 reagent at room temperature, 200uL EVs-free FBS was used to stop the staining, the labeled EVs were obtained by centrifugation (120 000xg, 70min, 4°C) and resuspended in 100uL PBS. The concentration and size distribution of EVs were measured by Nano-particle analysis (ZetaView, PMX 110, Germany), Western blot assay was used for characterization of EVs related protein biomarkers. The microstructure of EVs was observed by a transmission electron microscope (Hitachi, Tokyo, Japan) using 1% (v/v) urayl acetate staining.

### EVs uptake assays

HCT-8 and SW480 cells were seeded and reached 50% confluence in a six-well plate, 100uL PKH67 labeled EVs were added and continued to incubate for 12h. The cells were then fixed by 4% paraformaldehyde, followed by DAPI and Phalloidin staining, images were captured using confocal microscope (Olympus, Fluoview FV1200, Tokyo, Japan).

### EVs mild digestion assays

RNase and proteinase combined with Triton X-100 were used to eliminate EVs-free and intra-EVs RNAs and proteins. EVs were primarily treated with 100 μg/mL proteinase K (TIANGEN, cat. RT403, Beijing, China) at 37°C for 30min, followed by 10μg/mL RNase A (TIANGEN, cat. RT405, Beijing, China) at 37°C for 15min, additionally 0.1% Triton X-100 was added between RNase A and proteinase K treatment 20.

### Cell proliferation assays

MTS assay: After seeding SW480 and HCT-8 cells in 96-well plate, 20 μL MTS reagent (Promega, cat. G3581, WI, USA) was added into per well at the time point of 0, 24, 48, 72 and 96h, the absorbance at 490 nm was measured by microplate reader (SpectraMax M3, Molecular Devices, San Jose, CA) after incubation for 2h at 37°C.

EDU assay: 50nM EDU reagent (RiboBioInc, cat. C00003, Guangzhou, China) was added into 24-well plate seeded with SW480 and HCT-8 cells and incubated for 2h at 37°C, then fixed with 4% PFA, treated with glycine, followed by Apollo and Hoechst staining under manufacturer's instructions, representative images were captured under fluorescence microscope.

### Cell motility assays

The migration and invasion abilities of SW480 and HCT-8 were measured using Transwell migration chamber (Corning, cat. 353097, NC, USA) and Corning Matrigel Invasion chamber (Corning, cat. 354480, MA, USA) in a 24-well plate. Briefly, the basolateral chambers were added with 750uL complete medium and restored to 37°C before cell seeding, cells after transfection or incubating with M0/M2-EVs were added into the apical chamber with FBS-free medium, after 48h of incubation, the chamber were fixed and stained with methanol containing 0.1% crystal violet. Cells on the surface of upper chamber were wiped off and the membranes were gently embedded in resin and observed under inverted microscope (Olympus, CKX53, Tokyo, Japan), images of at least three random field were captured for each chamber.

### Extraction of RNA and qRT-PCR assays

EVs RNA was isolated and purified using miRNeasy^®^ Kit (Qiagen, cat. 217004, Hilden, Germany) under manufacturer's instructions. RNA of cultured cells was isolated by TRIzol Reagent (Thermo Fisher, cat. 15596026, Waltham, MA, USA). RNA was further processed by reverse transcriptase kit (TakaRa, cat. RR036A-1, Tokyo, Japan) to obtain cDNA. qPCR was conducted with PowerUp^TM^ SYBR Master Mix reagents (Life Technologies, cat. A25742, CA, USA), expression level of mRNA was analyzed using 2^-ΔΔCT^ standardized to GAPDH or U6. The sequences of primers were listed in Supplementary Table 1.

### Western blot assays

Protein obtained from each sample were quantified using Pierce BCA Protein Assay Kit (Thermo Fisher, cat. 23225, Waltham, MA, USA), protein samples with equal amount were loaded on 10% SDS-PAGE gel, the gel was then transferred to a PVDF membrane (Millipore, cat. IPVH00010, MA, USA) after separation, followed by blocking in 5% fat-free milk, then the membranes were submerged under primary antibodies overnight at 4°C, followed by specific secondary antibodies for 2h at 37°C, proteins bands were finally visualized using Gel imaging system (Bio-Rad, ChemiDoc XRS+, CA, USA). Primary antibodies used in our study were listed in Supplementary Table 2.

### Transfection of mimics, inhibitor and plasmids

Mimics and inhibitor of miR-186-5p were bought from Shanghai GenePharma Co, Ltd, the sequence of mimics is CAAAGAAUUCUCCUUUUGGGCU, sequence of mimics NC is UUGUACUACACAAAAGUACUG, the sequence of inhibitor is AGCCCAAAAGGAGAAUUCUUUG, the sequence of inhibitor NC is CAGUACUUUUGUGUAGUACAA. Plasmids encoding DLC1 were bought from Changsha Youbio Co, Ltd, the full length of DLC1 ORF sequence was cloned into pcDNA 3.1(+) vector. Transfection of mimics or inhibitor, mimics and plasmid co-transfection were performed with SiTran 2.0 reagent (OriGene Technologies, cat. TT320002, Beijing, China) under manufacturer's instructions. The transfection efficiency was assessed 48h for PCR and 72h for Western blot.

### Coculture of M2 macrophages and colon cancer cells

M2 macrophages were seeded in six-well plate, transfected with Cy3-labeled miR-186-5p mimics (Shanghai GenePharma Co, Ltd) using SiTran 2.0 reagent (OriGene Technologies, cat. TT320002, Beijing, China), and then cocultured with HCT-8 and SW480 using an upper chamber of 0.4μm pore size membrane (Corning, REF 3412, NC, USA). Images of HCT-8 and SW480 were taken by confocal microscope (Olympus, Fluoview FV1200, Tokyo, Japan) 24h post coculture.

### RNA pulldown assays

3' Biotin-labeled miR-186-5p mimics were bought from Shanghai GenePharma Co, Ltd. 3'Biotin-labeled mimics and its NC were transfected into the SW480 and HCT-8 using SiTran 2.0 reagent (OriGene Technologies, cat. TT320002, Beijing, China). Cells were rinsed and lysed 48h post transfection using DZ lysis buffer (Coolaber, cat. DZSL0770, Beijing, China) containing 5nM DTT, 1X proteinase inhibitor cocktail (Roche, cat. 04693132001, Basel, Switzerland) and RNase inhibitor (TakaRa, cat. 2313A, Tokyo, Japan), cell lysates were centrifuged and the supernatant was added with NaCl to reach concentration of 1M. Dynabeads™ MyOne™ Streptavidin C1 (Thermo Fisher, cat. 65001, Waltham, USA) were added to block solution containing Bovine serum albumin (Thermo Fisher, cat. 11020039, Waltham, USA) and Yeast tRNA (Thermo Fisher, cat. 15401029, Waltham, USA) before mixed with cell lysates for 2.5h at 4°C 21. Then the beads were rinsed by DZ washing buffer (Coolaber, cat. DZSL0770, Beijing, China), which is continued by RNA extraction using miRNeasy^®^ Kit (Qiagen, cat. 217004, Hilden, Germany). qRT-PCR were performed with extracted RNA as previously described.

### Luciferase reporter assays

Mutant and wild type sequence of DLC1 3'untranslated region(3'UTR) were cloned into the pmirGLO vector bought from Changsha Youbio Co, Ltd. HCT-8 and SW480 cells were co-transfected with miR-186-5p mimics/NC and pmirGLO DLC1 WT/MUT using SiTran 2.0 reagent (OriGene Technologies, cat. TT320002, Beijing, China). The luciferase activity of both renilla and firefly were analyzed with Dual-Luciferase^®^ Reporter Assay system (Promega, cat. E1910, WI, USA) after 48h post transfection.

### Statistical analysis

Graphpad 8.0 was used for data analysis. Data with at least three independent experiments were presented as mean ± SEM. Independent-sample Student's t test was used to analyze the differences between two unpaired groups, and differences between multiple groups were analyzed with one-way ANOVA. *P*<0.05 was considered as statistically significant.

## Results

### M2-EVs promoted the growth and motility of CC cells

**Figure 1:**
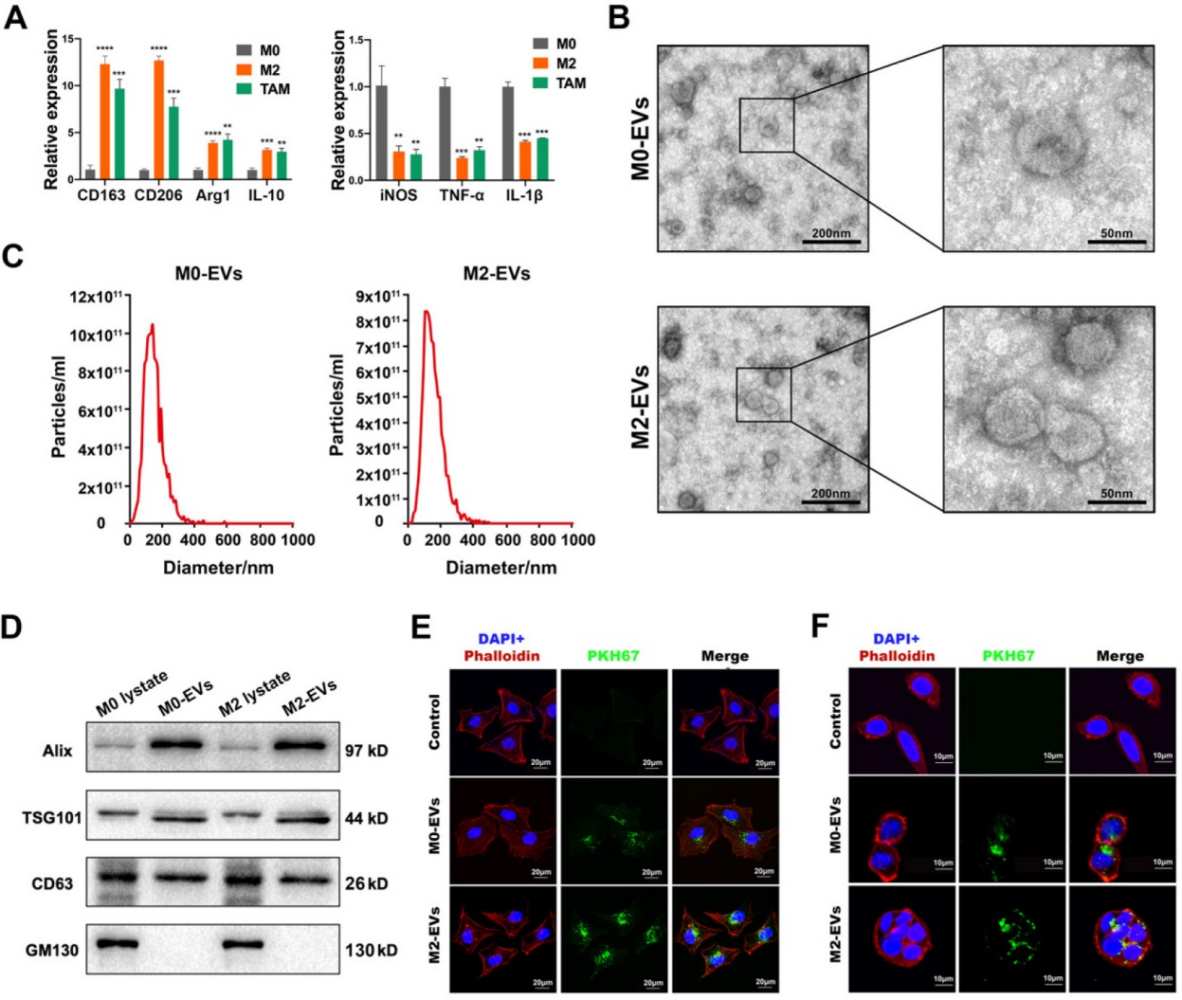
**Characterization of macrophages and their EVs. (A)** RT-PCR was used to compare the expression level of M2 and M1 macrophage biomarkers among M0 macrophage, IL-4 and IL-13 induced M2 macrophage and SW480-condition medium stimulated macrophages (**p<0.01, ***p<0.001, ****p<0.0001, compared to M0 macrophage group). **(B)** Morphological images of M0 and M2 macrophage derived EVs were captured by transmission electron microscope. (left scale bar = 200nm, right scale bar = 50nm).** (C)** Nano-particle analysis was used to analyze the particle size distribution of M0 and M2 macrophage derived EVs. **(D)** Western blot assay was used to detect protein biomarkers of EVs. **(E)** PKH67 labeled M0 and M2 macrophage derived EVs were internalized by SW480 (scale bar = 20μm). **(F)** PKH67 labeled M0 and M2 macrophage derived EVs were internalized by HCT-8 (scale bar = 10μm).

The differentiation of the M0 macrophage to M2 phenotype was induced by a combined treatment of IL-13 and IL-4, while macrophages stimulated by the conditioned medium of SW480 were used to simulate TAMs. The results indicated that IL-13 and IL-4 induced a higher level of M2 biomarkers such as CD163, CD206, Arg1 and IL-10 (Fig. 1A, left panel), and a lower level of M1 biomarkers such as iNOS, TNF-α, and IL-1β (Fig. 1A, right panel), suggesting a successful differentiation of M2 macrophage. The conditioned medium of SW480 also induced a similar pattern of biomarkers to M2 macrophages (Fig. 1A), indicating a successful simulation of TAM.

M0 and M2 macrophage-derived EVs were isolated and purified by ultracentrifugation. Both M0 and M2-derived EVs were clearly presented as a typical “cup-like” structure under a transmission electron microscope (Fig. 1B). NTA analysis indicated that those EVs were nanoparticles with a diameter around 140 nm (Fig. 1C). Western Blot analysis confirmed those EVs were positive for EVs biomarkers (Alix, TSG101, and CD63), and negative for cytoplasmic biomarker GM130 (Fig. 1D). No significant difference between M0-EVs and M2-EVs was identified in those characterization assays. EVs uptake assays were performed to ensure the internalization of those EVs by cancer cells. Our results suggested that both PKH67 labeled (green fluorescence) M0 and M2-EVs were visualized within SW480 and HCT-8, suggesting that those macrophage-derived EVs were successfully internalized by the cancer cells (Fig. 1E-F).

**Figure 2:**
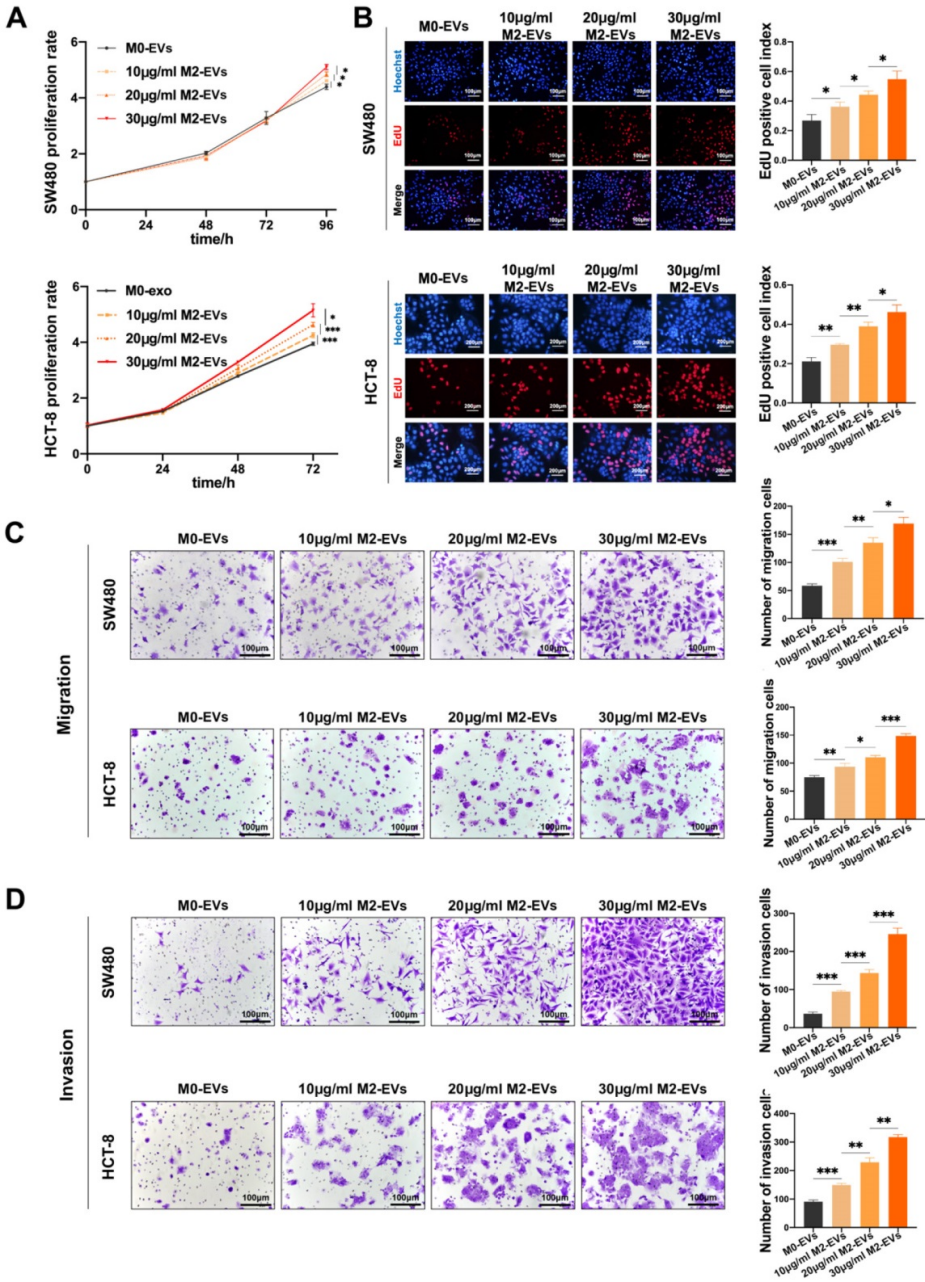
**M2-EVs promoted the growth and motility of CC. (A)** MTS assay was used to detect the proliferation of CC cells under M2-EVs with different concentration (*p<0.05, ***p<0.001).** (B)** EdU assay was used to detect the proliferation of SW480 (scale bar = 100μm) and HCT-8 (scale bar = 200μm) (*p<0.05, **p<0.01). **(C-D)** Transwell migration and invasion assay were used to detect the motility of CC cells stimulated by M2-EVs (scale bar = 100μm) (*p<0.05, **p<0.01, ***p<0.001).

The growth and motility of CC cells were measured after treated with M0-EVs and M2-EVs. The MTS assays indicated that the proliferation ability of both SW480 and HCT-8 were significantly enhanced by M2-EVs in a dose-dependent manner (Fig. 2A), which is consistent with EdU assays (Fig. 2B). Then we performed Transwell assays to verify the effect of M2-EVs on the motility of CC cells. As shown in Fig. 2C-D, the number of both migrated and invaded cells increased significantly after stimulated by M2-EVs. Our results confirmed that M2-EVs boosted the growth and motility of CC cells in a dose-dependent manner.

### The tumor-promoting effects of M2-EVs depended on their enclosed molecules

**Figure 3:**
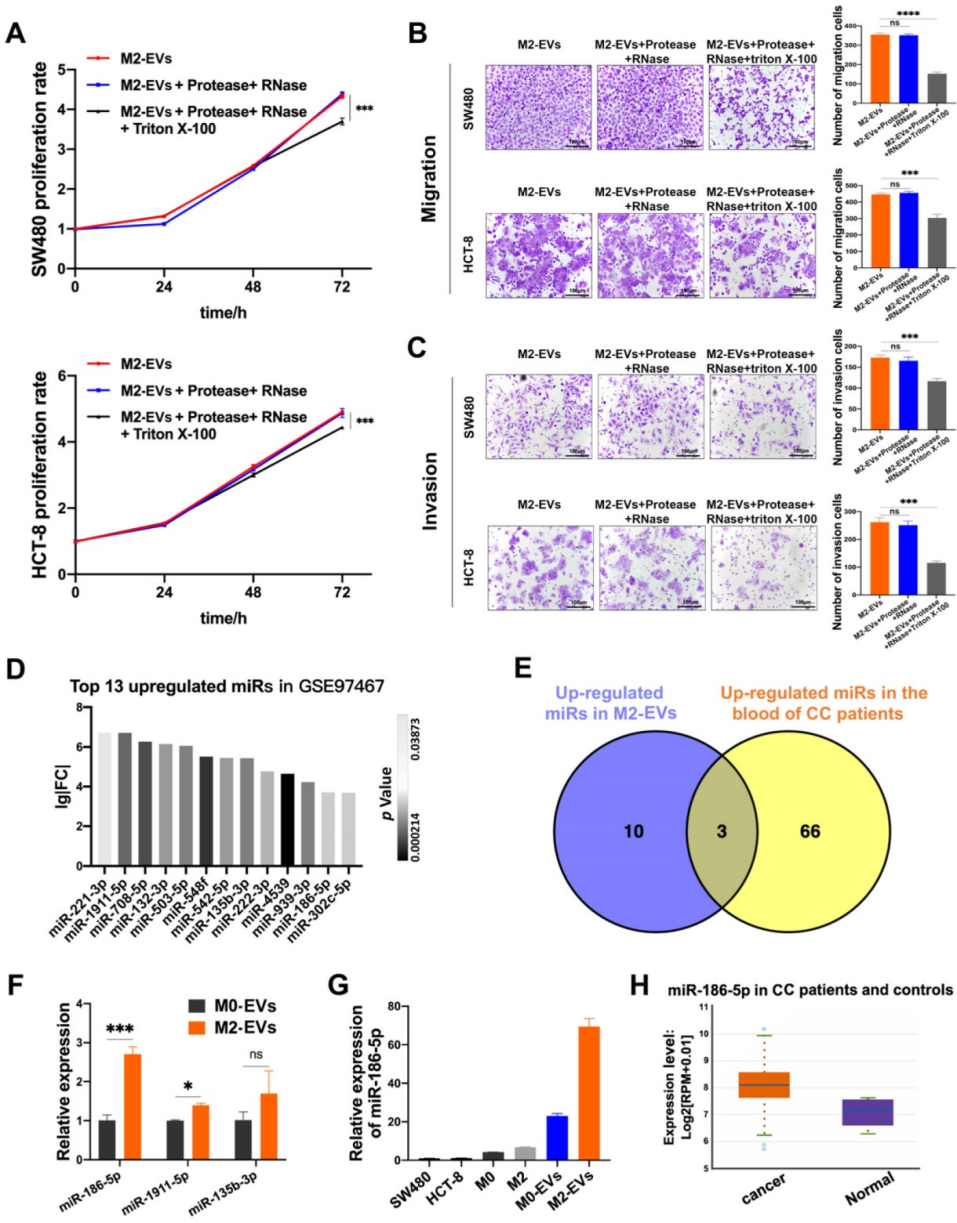
**The tumor-promoting effects of M2-EVs depended on their enclosed molecules. (A)** MTS assay was used to detect the proliferation of CC cells incubated in processed M2-EVs (***p<0.001). **(B-C)** Transwell migration and invasion assay were used to detect the motility of CC cells stimulated by processed M2-EVs (scale bar = 100μm) (***p<0.001, ****p<0.0001).** (D)** 13 upregulated microRNAs in GSE97467 (Geo DataSet) were illustrated in the histogram. **(E)** The intersection of GSE97467 and GSE61741 was presented in venn graph. **(F)** RT-PCR was used to determine the expression level of three candidate microRNAs in macrophages derived EVs (*p<0.05, ***p<0.001). **(G)** RT-PCR was used to detect the relative expression level of miR-186-5p between CC cells, macrophages and macrophage derived EVs. **(H)** The relative expression profiles of miR-186-5p in CC patients and healthy controls were analyzed using StarBase database, box on the left presents CC patients and the right box presents healthy controls.

M2-EVs were treated with RNase and protease to eliminate both co-precipitated free-floating RNA-protein complexes (*e.g.,* Agos and their binding RNAs) and RNAs/proteins located on the superficies of EVs, while the synchronous Triton X-100 treatment was used to degrade all the RNA and protein. M2-EVs treated with RNase and protease exhibited the same promoting effects on proliferation as compared to naïve M2-EVs, but the synchronous treatment of Triton X-100 significantly abolished their growth-promoting effects in both SW480 and HCT-8 (Fig. 3A). Similarly, as shown in Fig. 3B-C, the treatment with Triton X-100 also attenuates the promoting effects of M2-EVs on the motility of CC cells. Therefore, the EVs-enclosed molecules rather than free-floating RNAs or proteins were responsible for the tumor-promoting effects of M2-EVs.

Then we investigated the possible candidates for tumor-promoting effects of M2-EVs by re-analyzing the public available miRNA GEO datasets. MiRNA profiling data of GSE97467 (miRNA microarrays of M0 and M2 macrophage-derived EVs) was analyzed by GEO2R. 13 microRNAs were significantly upregulated in M2-EVs compared with M0-EVs (Fig. 3D, P<0.05, lg|FC|≥2). Then GSE61741 (non-coding RNA profiling arrays comparing the plasma of CC patients and healthy controls) was chosen to verify the presence of candidate miRNAs in blood, and three up-regulated microRNA (miR-135b-3p, miR-1911-5p, and miR-186-5p) were further identified as potential circulating biomarkers (Fig. 3E). Next, the results of RT-qPCR indicated that miR-186-5p was the most abundant miRNA in M2-EVs among the three candidates (Fig. 3F). Furthermore, we confirmed that miR-186-5p expression level is relatively low in macrophages and CC cells, but it was enriched in macrophage-derived EVs, especially in M2-EVs (Fig. 3G). Additionally, we used the Starbase database to analyze the expression profile of miR-186-5p in CC patients, which is at a higher level compared with healthy controls (Fig. 3H), indicating potential tumor-promoting roles of miR-186-5p.

**Figure 4:**
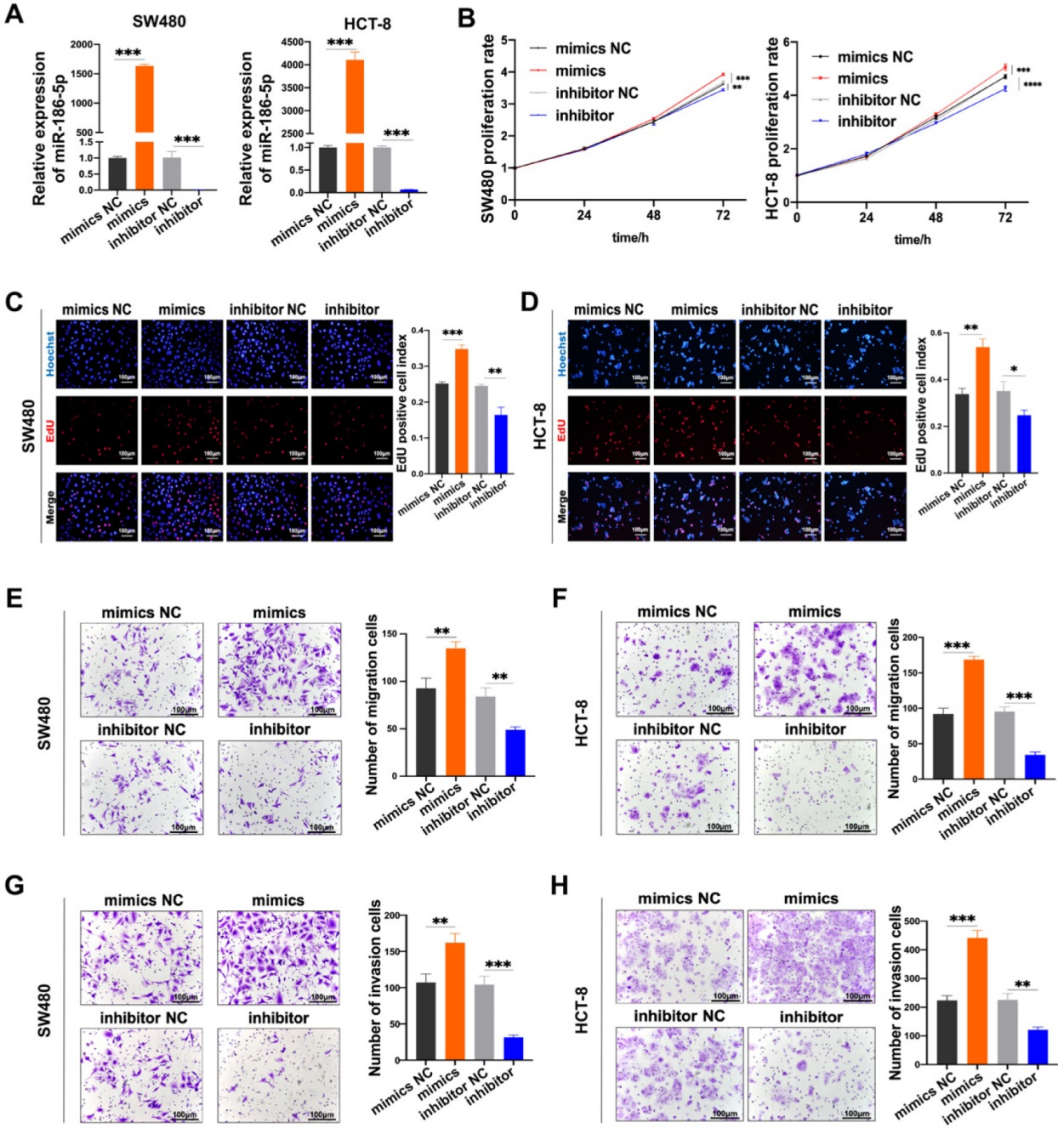
** MiR-186-5p promotes the growth and motility of CC. (A)** RT-PCR was used to detect the expression of miR-186-5p after transfected with mimics and inhibitor in CC cells (***p<0.001). **(B-D)** MTS and EdU assay were used to detect the proliferation of CC cells transfected with miR-186-5p mimics and inhibitor (*p<0.05, **p<0.01, ***p<0.001, ****p<0.0001). **(E-H)** Transwell migration and invasion assay were used to assess the motility of CC cells transfected with mimics and inhibitor (scale bar = 100μm) (**p<0.01, ***p<0.001).

Then we investigated the biological effects of miR-186-5p in CC cells. Firstly, we confirmed the expression of miR-186-5p was successfully up-regulated by its mimics while down-regulated inhibitor (Fig. 4A). Then the growth and motility of cancer cells were assessed after transfected with those mimics and inhibitor. The proliferation of both SW480 and HCT-8 cells significantly increased by mimics, while significantly decreased by inhibitor (Fig. 4B-D). Similarly, as shown in Fig. 4E-H, both migration and invasion abilities of CC cells were significantly enhanced by mimics, while attenuated by inhibitor. Taken all together, we confirmed that M2-EVs derived miR-186-5p could enhance the growth and motility of CC cells.

### MiR-186-5p was responsible for the tumor-promoting effects of M2-EVs

**Figure 5:**
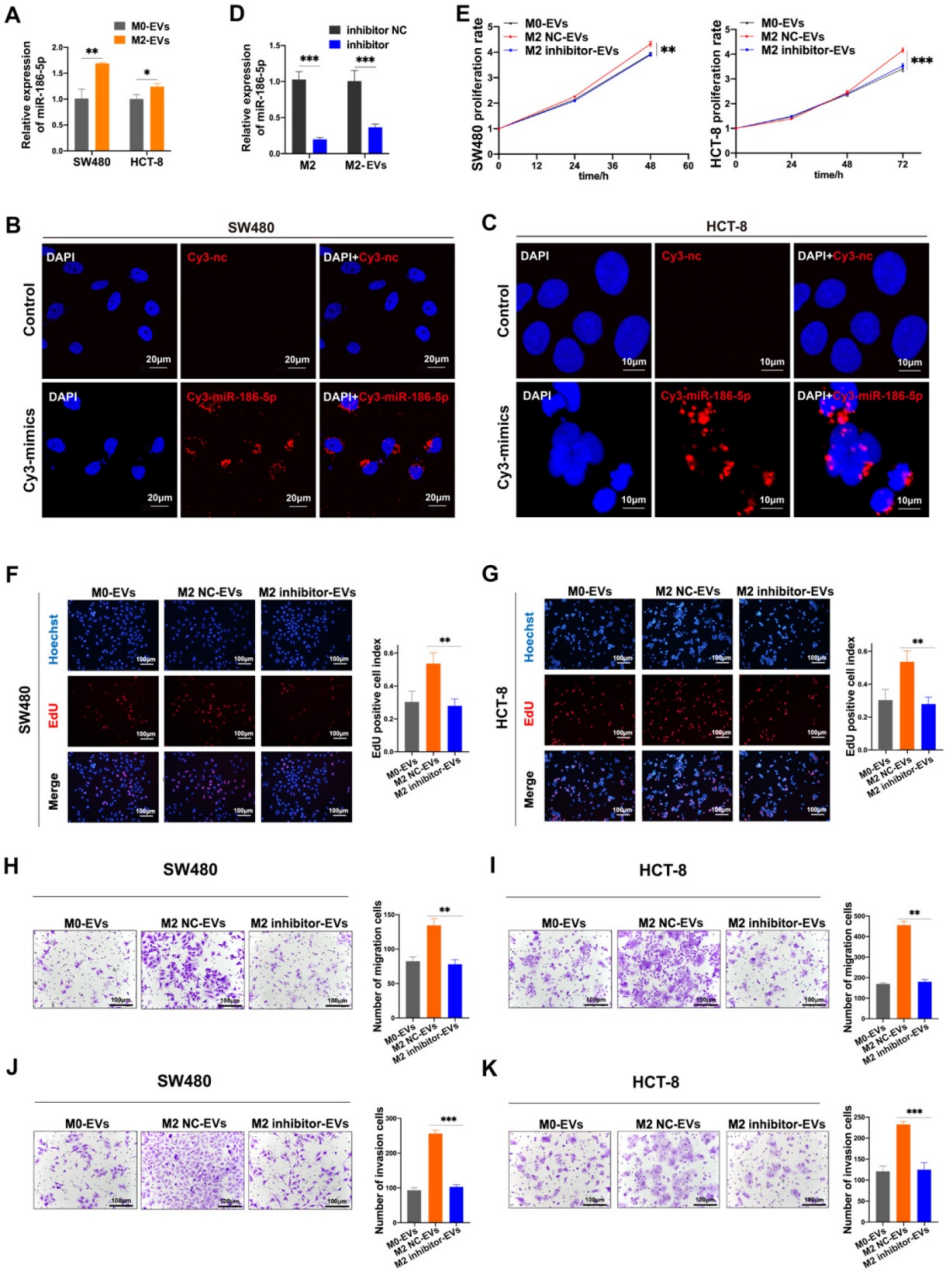
** MiR-186-5p was responsible for the promotion effects of M2-EVs. (A)** RT-PCR was used to detect the expression level of miR-186-5p in CC cells stimulated by M2-EVs (*p<0.05, **p<0.01). **(B-C)** Images of SW480 (scale bar = 20μm) and HCT-8 (scale bar = 10μm) were taken after cocultured with M2 macrophages transfected with Cy3-labeled miR-186-5p mimics (Cy3-mimics). **(D)** RT-PCR was used to detect the expression of miR-186-5p in M2 macrophage and M2-EVs after transfected with its inhibitor (***p<0.001). **(E-G)** MTS and EdU assay was used to detect the proliferation rates of CC cells stimulated by M2-EVs with reduced miR-186-5p level (scale bar = 100μm) (**p<0.01, ***p<0.001). **(H-K)** Transwell migration and invasion assay was used to determine the motility of CC cells stimulated by M2-EVs with reduced miR-186-5p level. (scale bar = 100μm) (**p<0.01, ***p<0.001, ****p<0.0001).

Given that miR-186-5p was enriched in M2-EVs and both of them exhibited tumor-promoting effects on CC cells, the tumor-promoting effects of M2-EVs could be dependent on miR-186-5p. The expression level of miR-186-5p in cancer cells was significantly increased after treated with M2-EVs (Fig. 5A). Also, red florescence was detected in both HCT-8 and SW480 after cocultured with M2 macrophages transfected with Cy3-labeled miR-186-5p mimics (Fig. 5B-C), suggesting that miR-186-5p could be successfully delivered into CC cells via M2-EVs. Then we transfected M2 macrophages with inhibitor to knock down the expression of miR-186-5p in M2 macrophage-derived EVs (M2 inhibitor-EVs), and the expression of miR-186-5p in M2 inhibitor-EVs was also significantly decreased (Fig. 5D). As shown in Fig. 5E-G, M2 inhibitor-EVs exhibited significantly decreased tumor-promoting effects on proliferation as compared to naïve M2-EVs. Similarly, the motility of both cell lines enhanced by M2-EVs were also largely attenuated by transfecting M2 macrophages with miR-186-5p inhibitor (Fig. 5H-K). Collectively, we proved that miR-186-5p is responsible for the promoting effects of M2-EVs.

### MiR-186-5p down-regulated DLC1 by directly binding to its 3'UTR

**Figure 6:**
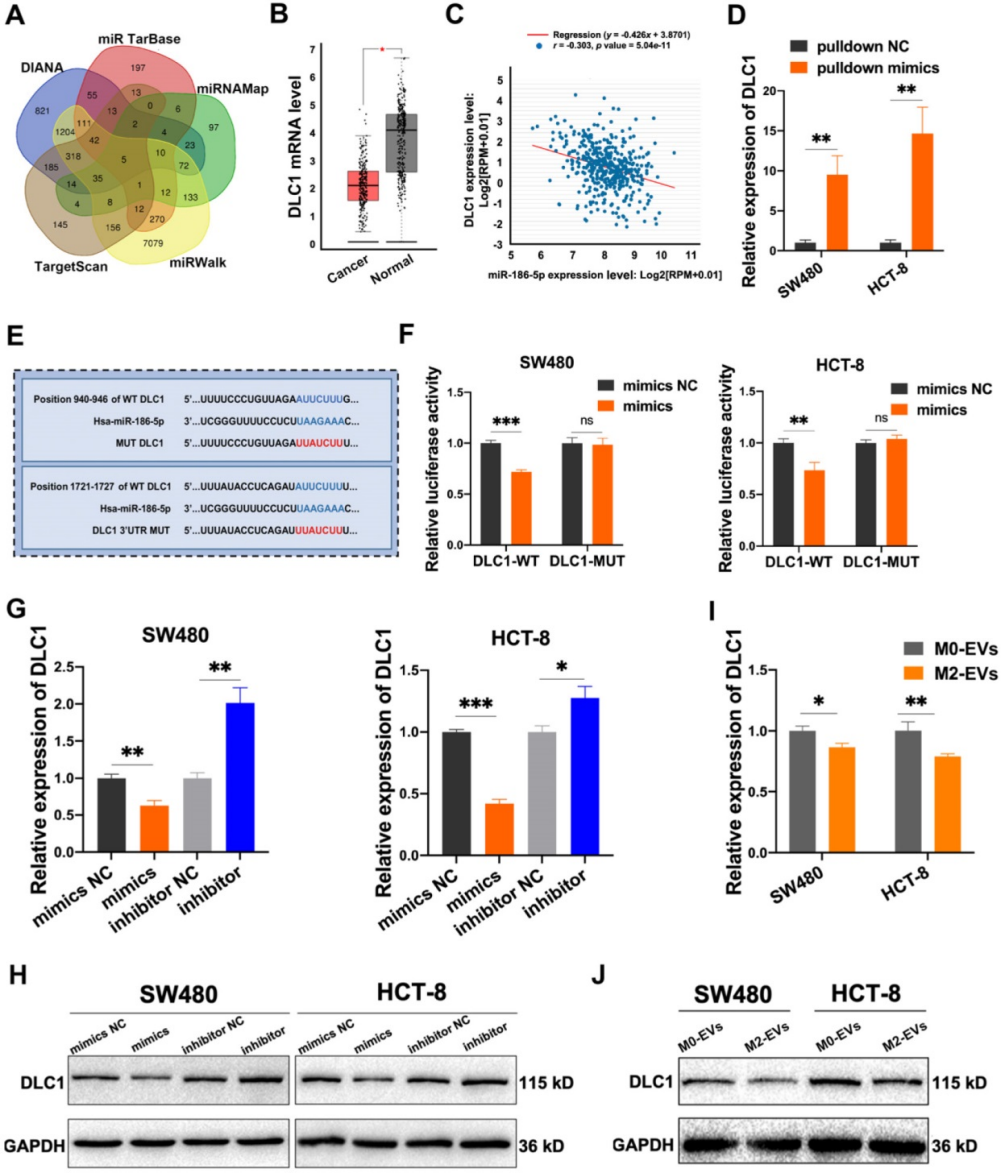
**MiR-186-5p inhibited DLC1 expression by combining with its 3'UTR. (A)** The target genes of miR-186-5p presented in venn map were predicted by TargetScan, DIANA, miRTarBase, miRNAMap and miRWalk. **(B)** The relative expression level of DLC1 in CC patients and healthy control was presented using GEPIA database, red box indicates CC patients and the other indicates healthy control (*p<0.05). **(C)** The expression level between miR-186-5p and DLC1 in CC patients was performed by StarBase database. **(D)** The combination of miR-186-5p and DLC1 mRNA was verified by RNA pulldown assay (**p<0.01). **(E)** The binding sites of miR-186-5p and DLC1 3'UTR were predicted using TargetScan. **(F)** Luciferase reporter assay was used to confirm the combination of miR-186-5p and DLC1 3'UTR (**p<0.01, ***p<0.001). **(G-H)** RT-PCR and Western blot was used to determine the mRNA and protein level of DLC1 in CC cells after mimics and inhibitor transfection (*p<0.05, **p<0.01, ***p<0.001). **(I-J)** RT-PCR and Western blot was used to determine the mRNA and protein level of DLC1 in CC cells after stimulated by M0/M2-EVs (*p<0.05, **p<0.01).

Five online miRNA-targets prediction tools including TargetScan, DIANA, miRTarbase, miRWalk and miRNAMap were used to explore the potential targets of miR-186-5p. Only 5 genes were obtained in the intersection of 5 databases (Fig. 6A), among which DLC1, also named Delete in liver cancer-1, was a metastasis suppressor in hepatocellular carcinoma. The analysis of the GEPIA database also identified DLC1 as a significantly down-regulated gene in CC patients (Fig. 6B). Moreover, we found a negative correlation of DLC1 mRNA level and miR-186-5p in CC patients (Fig. 6C), suggesting DLC1 was a possible target gene of miR-186-5p. To test whether miR-186-5p could bind to DLC1 mRNA, we used 3' biotin-labeled miR-186-5p mimics for RNA pulldown assay. As shown in Fig. 6D, DLC1 mRNA pulled-down by biotin-labeled mimics was significantly higher as compared with biotin-labeled mimics NC, indicating miR-186-5p could combine with DLC1 mRNA. The dual-luciferase reporter assay also suggested that the luciferase activity of wild-type but not mutated 3'UTR sequence of DLC1 mRNA was significantly reduced by miR-186-5p mimics (Fig. 6E-F), indicating miR-186-5p could bind to the DLC1 promoter. Next, we confirmed that both mRNA and protein expression levels of DLC1 were decreased by miR-186-5p mimics while up-regulated by inhibitor in both CC cells (Fig. 6G-H). Considering that miR-186-5p is enriched in M2-EVs, we also tested whether M2-EVs could regulate DLC1 expression in cancer cells. Results in Fig. 6 I and J suggested that both mRNA and protein expression levels of CC cells were significantly decreased after treated with M2-EVs, which was consistent with transfection of miR-186-5p mimics. Therefore, here we concluded that miR-186-5p down-regulated DLC1 by targeting its 3'UTR.

### MiR-186-5p enhanced the growth and motility of CC by targeting DLC1

**Figure 7:**
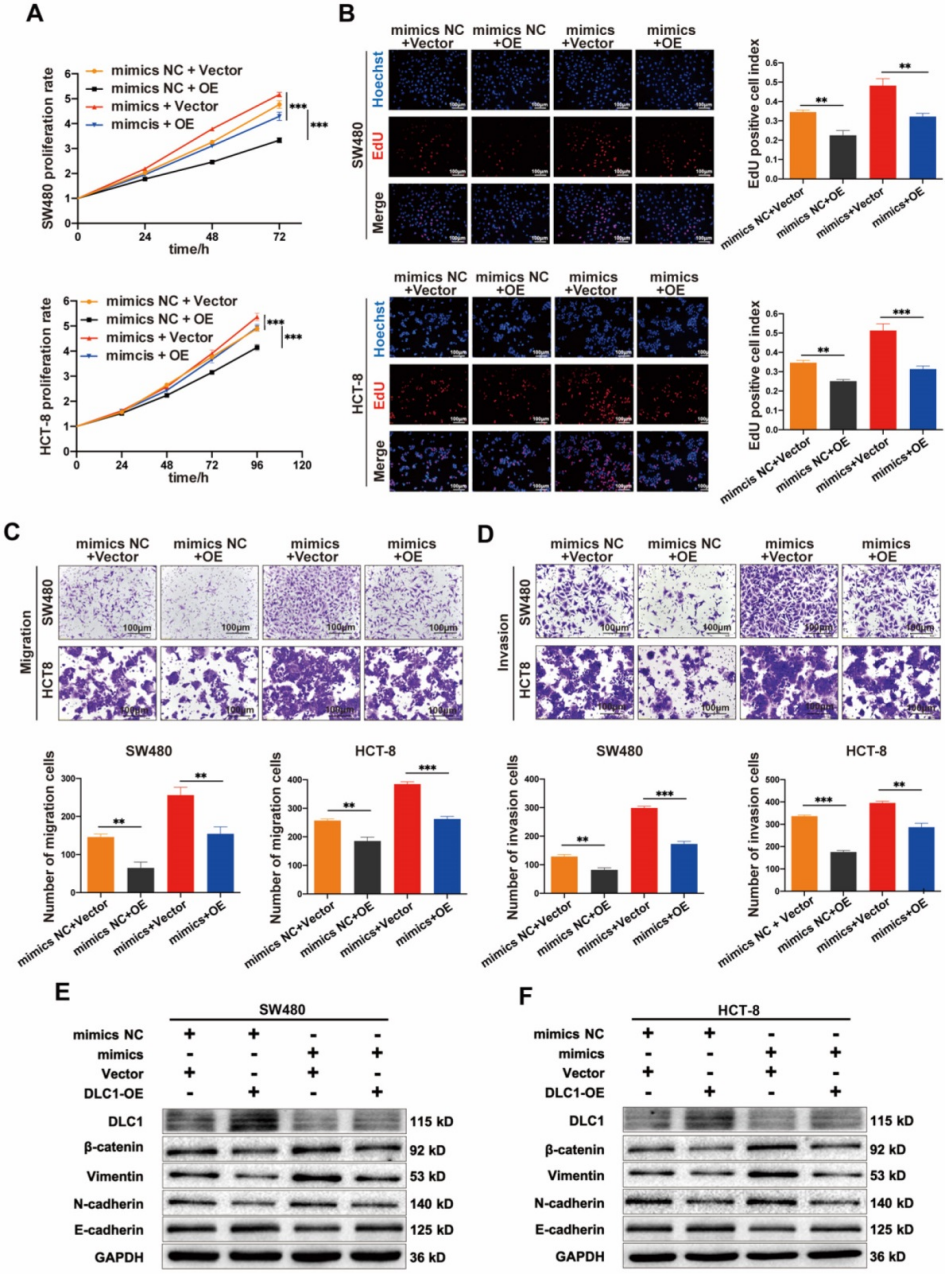
**MiR-186-5p enhanced the growth and motility of CC by inhibiting DLC1. (A-B)** MTS and EdU assay were used to detect the proliferation rates of CC cells co-transfected with mimics and DLC1 overexpression plasmid (**p<0.01, ***p<0.001). **(C-D)** Transwell migration and invasion assay were used to assess the motility of CC cells after co-transfection (scale bar = 100μm) (**p<0.01, ***p<0.001).** (E-F)** Western blot assay was used to detect expression level of DLC1, β-catenin and epithelial mesenchymal transition (EMT) related proteins in CC cells after co-transfection.

Then we tested whether the tumor-promoting effects of miR-186-5p depend on its regulation on DLC1. Shown in Fig. 7A-D, the growth and motility of CC cells were inhibited by DLC1 overexpression significantly, and the promoting effects of miR-186-5p mimics could also be largely reversed by DLC1 overexpression, indicating DLC1 was crucial in the tumor-promoting effects of miR-186-5p. It is known that DLC1 inhibits cell growth and motility via downstream pathways such as Wnt/β-catenin and NF-κB signaling. Thus, here we also assessed protein levels of β-catenin and EMT-related biomarkers. Results in Fig. 7E-F confirmed the successful overexpression of DLC1 at the protein level, and the down-regulated DLC1 under miR-186-5p mimics could be restored by DLC1 overexpression. Furthermore, DLC1 overexpression down-regulated β-catenin level, was resulting in the decrease of EMT biomarkers Vimentin, N-cadherin and increase of E-cadherin. While miR-186-5p mimics activated β-catenin signaling pathway, promoting EMT process, which could also be reversed by DLC1 overexpression. Collectively, we concluded that miR-186-5p enhanced the growth and motility of CC cells by inhibiting DLC1.

## Discussion

Increasing evidence demonstrated that TAMs are a mixture composed of both M1 and M2 macrophages 14. Wu TH *et al*. found that CC cell-derived condition medium induced THP-1 cells into an M1/M2 mixed population 22, which was consistent with the findings reported by other groups 23. Here we used interleukin-induced M2 macrophages instead of tumor tissue-isolated TAMs to investigate the underlying mechanism of tumor-promoting M2-EVs subpopulation to avoid the confounding factors brought by the heterogeneity of tumor tissue-isolated TAMs. The main finding that M2-EVs promoted the growth and motility of cancer cells is consistent with previous reports based on TAMs, further confirmed that the dominant subpopulation of TAMs is M2 macrophage. However, here we first reported that the EVs-enclosed miR-186-5p was responsible for the tumor-promoting effect of M2-EVs. The effacement of the role of miR-186-5p in TAM-EVs would be a result of the adulteration of M1 macrophages in the studies performed with tumor tissue-isolated TAMs.

Recently, there is no ideal method for EVs isolation. Different approaches (*e.g.,* ultracentrifugation, size exclusion chromatography, density gradients centrifugation, and polymer-based precipitation) have been developed, and researchers always need to trade off recovery against purity 24. The obtained EVs fractions were inevitable to be contaminated with unexpected proteins co-isolated 25 and free-floating nucleic acids adsorbed on the surface of EVs 26, 27. The effects of these dopants should be evaluated and considered to clarify the function of isolated EVs. Unfortunately, most of the studies on TAM-EVs ignored this issue. According to the latest guideline in EVs research, strategies such as mild digestion is advised to achieve putative active components 24. Here we use RNase and protease to digest RNAs and proteins on the surface of EVs as well as the free-floating ones without breaking the integrity of lipid bilayers. We found this mild digestion had no effect on the biological functions of M2-EVs, demonstrating only the enclosed molecules inside lipid bilayers were crucial for their tumor-promoting roles.

Numerous microRNAs were found to take part in cancer development, and many of them have been demonstrated to be delivered to recipient cells via EVs. MiR-186-5p was up-regulated in different tumors and contributed to the development of prostate cancer 28, lung cancer 29, and colorectal cancer 30. Here we found the increased miR-186-5p level was associated with enhanced growth and motility in CC, which was consistent with previous studies. Remarkably, we also proved the enrichment of miR-186-5p in M2-EVs and confirmed that miR-186-5p could be delivered from M2 macrophages to CC cells via EVs for the first time. However, other microRNAs enriched in M2-EVs could exhibit similar tumor promoting effects through the delivery of EVs. MiR-221-3p was reported as an oncogene in hepatocellular carcinoma 31 and non-small cell lung cancer 32, which was also found to function though EVs in the promotion of cervical squamous cell carcinoma 33, 34. Such oncogenic microRNAs could also serve as crucial effectors for the tumor promoting abilities of M2-EVs, which is worthy to be elucidated in the future.

In this article, we proposed 5 consensus possible targets of miR-186-5p and validated DLC1 in molecular biological function assays. However, other genes are also possible to be manipulated by EVs-miR-186-5p in cancer cells. For example, STAG2, as one of the other 4 predicted consensus genes, regulates the procedure of sister chromatids separation. Deactivated STAG2 resulted in aneuploidy, which was observed in melanoma, Ewing's sarcoma and glioblastoma 35. Another gene named PHIP was found to be related to glial oncogenesis 36, and was considered as possible tumor suppressor in a murine medulloblastoma model 37. These potential target genes are also worth investigating in the future.

As we proved that M2-EVs miR-186-5p promoted CC progression via targeting DLC1, some intervention approaches against this process could be proposed. Molecules localized on the surface of EVs were proved to be involved in their uptake by different target cells. Hoshino A. *et al.* found sealing different subtypes of integrin decreased EVs uptake by specific organs 38. Laulagnier K. *et al.* demonstrated that the glial cells took up CD63+ EVs but not CD63- EVs 39. Interruption of the internalization of M2-EVs by cancer cells would largely abolish the tumor-promoting activity of M2 in TME, which would inspire researchers to develop new tumor-suppressing approaches targeting TME.

## Conclusion

We proposed a new mechanism that M2 macrophage-derived EVs delivering miR-186-5p down-regulated DLC1, therefore enhanced the growth and motility of CC cells by activating the β-catenin pathway. This study not only provided a new mechanism in intercellular communication between stromal cells and cancer cells but also expanded our understanding of the regulatory effects of M2 macrophage in TME.

## Supplementary Material

Supplementary tables.
