# Supplementary material for: M2 Macrophage Derived Extracellular Vesicle-Mediated Transfer of MiR-186-5p Promotes Colon Cancer Progression by Targeting DLC1
Source: Int J Biol Sci. 2022 Feb 7;18(4):1663–76. doi: 10.7150/ijbs.69405 (PMC8898350; doi:10.7150/ijbs.69405)
Supplement: Supplementary file 1 — Supplementary tables. [file ijbsv18p1663s1.pdf]

Supplementary Table 1. The sequence of primers used in RT-qPCR

Gene	Sequence (5' →3')	
GAPDH	F: GGAGCGAGATCCCTCCAAAAT	R: GGCTGTTGTCATACTTCTCATGG
U6	F: AAAGCAAATCATCGGACGACC	R: GTACAACACATTGTTTCCTCGGA
Arg1	F: TGGACAGACTAGGAATTGGCA	R: CCAGTCCGTCAACATCAAAACT
CD163	F: GCGGGAGAGTGGAAGTGAAAG	R: GTTACAAATCACAGAGACCGCT
CD206	F: GGGTTGCTATCACTCTCTATGC	R: TTTCTTGTCTGTTGCCGTAGTT
IL-10	F: GACTTTAAGGGTTACCTGGGTTG	R: TCACATGCGCCTTGATGTCTG
IL-1β	F: TTCGACACATGGGATAACGAGG	R: TTTTTGCTGTGAGTCCCGGAG
iNOS	F: TTCAGTATCACAACCTCAGCAAG	R: TGGACCTGCAAGTTAAAATCCC
DLC1	F: GCGTACCTGTGTCGCTTTAT	R: CTCCTCTGTGCAAACCTTTCT
miR-135b-3p	F: ACACTCCAGCTGGGATGTAGG GCTAAAAGC	R: TGGTGTCGTGGAGTCG
miR-1911-5p	F: ACACTCCAGCTGGGTGAGTACCG CCATGTCT	R: TGGTGTCGTGGAGTCG
miR-186-5p	F: ACACTCCAGCTGGGCAAAGAATT CTCCTTT	R: TGGTGTCGTGGAGTCG

Supplementary Table 2. Primary antibodies used in Western Blot

Primary antibody	Manufacturer	Dilution
anti-Alix	ProteinTech, 12422-1-AP, Wuhan, China	1:2000
anti-TSG101	Abcam, ab125011, MA, USA	1:1000
anti-CD63	Abcam, ab134045, MA, USA	1:1000
anti-GM130	ProteinTech, 11308-1-AP, Wuhan, China	1:2000
anti-GAPDH	ProteinTech, 60004-1-Ig, Wuhan, China	1:5000
anti-DLC1	Santa Cruz, sc-271915, CA, USA	1:500
anti-DLC1	ProteinTech, 66894-1-Ig, Wuhan, China	1:2000
anti-β-catenin	ProteinTech, 51067-2-AP, Wuhan, China	1:5000
anti-Vimentin	ProteinTech, 10366-1-AP, Wuhan, China	1:2000
anti-N-cadherin	ProteinTech, 22018-1-AP, Wuhan, China	1:2000
anti-E-cadherin	ProteinTech, 20874-1-AP, Wuhan, China	1:5000
